# Genome-wide analysis of the rice J-protein family: identification, genomic organization, and expression profiles under multiple stresses

**DOI:** 10.1007/s13205-019-1880-8

**Published:** 2019-09-06

**Authors:** Ying Luo, Baohua Fang, Weiping Wang, Ying Yang, Liqun Rao, Chao Zhang

**Affiliations:** 1 College of Bioscience and Biotechnology, Hunan Agricultural University, 410125 Changsha, China; 2 Key Laboratory of Indica Rice Genetics and Breeding in the Middle and Lower Reaches of Yangtze River Valley, Ministry of Agriculture, 410125 Changsha, China; 3 State Key Laboratory of Hybrid Rice, Hunan Hybrid Rice Research Center, 410125 Changsha, China; 4 College of Chemistry and Bioengineering, Hunan University of Science and Engineering, Yongzhou, China

**Keywords:** Rice, J-protein, Genome-wide analysis, Expression profile, Abiotic stress

## Abstract

J-proteins which function as molecular chaperone played critical roles in plant growth, development, and response to various environment stresses, but little was reported on this gene family in rice. Here, we identified 115 putative rice J-proteins and classified them into nine major clades (I–IX) according to their phylogenetic relationships. Gene-structure analysis revealed that each member of the same clade has same or similar exon–intron structure, and most rice J-protein genes of clade VII were intronless. Chromosomes mapping suggested that tandem duplication was occurred in evolution. Expression profile showed that the 61 rice J-protein genes were expressed in at least one tissue. The result implied that they could be involved in the process of rice growth and development. The RNA-sequencing data identified 96 differentially expressed genes, 59.38% (57/96), 67.71% (65/96), and 62.50% (60/96) genes were induced by heat stress, drought stress, and salt stress, respectively. The results indicated that J-protein genes could participated in rice response to different stresses. The findings in this study would provide a foundation for further analyzing the function of J-proteins in rice.

**Electronic supplementary material:**

The online version of this article (10.1007/s13205-019-1880-8) contains supplementary material, which is available to authorized users.

## Introduction

Plants, as sessile organisms, have to deal with complex environmental cues including a variety of stresses, such as high salt, extreme temperature, water deficiency, oxidative stress, chemical pollutants, and pathogens (Al-Whaibi 2011). Unlike animals, plants cannot change their sites to escape from the unfavorable conditions, and therefore, they have evolved with a spectrum of molecular mechanism that regulates their cellular proteome with the changing external environment (Kosová et al. 2011; Kurepa et al. 2009). When the expression of the genes is coding for heat shock proteins (Hsps) which are trigged by heat, as well as in other stresses, Hsps accumulate in the organism (Gupta et al. 2010; Lindquist and Craig 1988), and increased expression of these genes can enhance the heat tolerance of plants (Wang et al. 2018). Hsps have been classified into six groups, such as Hsp100, Hsp90, Hsp70, Hsp60, Hsp40/J-protein, and small Hsp (sHsp/Hsp20) based on their molecular weight (Georgopoulos and Welch 1993; Lindquist and Craig 1988). The Hsp40 family of molecular chaperones includes DnaJ, and this family is also designated the J-protein family. J-proteins have been often regarded as obligate partners of Hsp70s as neither Hsp70s, nor the J-proteins can work without each other (Tamadaddi and Sahi 2016). In the integrated model of protein surveillance system, J-proteins are the co-chaperones of Hsp70, and the molecular mechanism of the latter collaborates with Hsp100; thus, the activity of Hsp70 is regulated by J-proteins (Miot et al. 2011; Sielaff and Tsai 2010). In all the organisms studied so far, the number of Hsp40s is always more than the number of Hsp70s. For example, there are 22 Hsp40s and 14 Hsp70s in *Saccharomyces cerevisiae*, seven Hsp40s and three Hsp70s in *Escherichia coli*, 45 Hsp40s and 17 Hsp70s in human, 36 Hsp40s and 11 Hsp70s in *Drosophila melanogaster*, 118 Hsp40s and 18 Hsp70s in *Arabidopsis thaliana* (Craig and Marszalek 2017; Walsh et al. 2004). Therefore, a single Hsp70 may bind a diversity of J-proteins to perform protein folding, prevention of protein aggregation, translocation of proteins across membranes, targeting proteins towards degradation, and regulation of translation initiation.

J-proteins were originally characterized from *E. coli* as a 41-kDa Hsps (Georgopoulos et al. 1980). The J-domain, the defining feature of all J-proteins, is a compact tetrahelical domain of ~ 70 residues with a highly conserved and functionally critical histidine, proline, and aspartic acid tripeptide (HPD) motif (Verma et al. 2017). J-proteins are classified into three types based on the presence of specific conserved regions. Type A J-proteins are characterized by an N-terminal J-domain followed by a glycine/phenylalanine (G/F)-rich region, four repeats of the CxxCxGxG-type zinc-finger domain, and a C-terminal domain. Type B J-proteins are very similar to Type A J-proteins, except that they lack the CxxCxGxG-type zinc-finger domain. Type C J-proteins are the most diverse group, as they only carry the J-domain. The proteins that contain a J-like domain but lack the critical HPD tripeptide are classified as type D J-proteins (Kampinga and Craig 2010). Rice J-proteins have been classified into three classes (corresponding to types A–C) according to domain organization (Sarka et al. 2013). However, some recently reported rice J-proteins still lack identification and their phylogenetic relationships are unknown; in addition, the expressions of the gene coding for J-proteins under multiple stresses are unclear.

In plants, J-proteins have been localized to different subcellular compartments. In rice, for example, 63, 15, and 8 J-proteins have been localized to the cytoplasm, chloroplast, and mitochondrion, respectively (Walsh et al. 2004). In *A. thaliana*, six J-proteins were localized to the endoplasmic reticulum (ER) and 19 to the chloroplast (Chiu et al. 2013; Ohta et al. 2013; Yamamoto et al. 2011). Furthermore, J-proteins not only function as co-chaperones in various biological processes (Miernyk 2001), but also act as enzymes or epigenetic regulators (De et al. 1995; Richly et al. 2010). In *A. thaliana*, the farnesylated J2 and J3 associate with AGO1 in membrane fractions in a manner that involves protein farnesylation, and also influences the distribution of miRNA between polysome-bound and unbound fraction (Sjögren et al. 2018); the J-proteins embryo sac development arrest 3 (EDA3) and *thermosensitive male sterile 1* (*TMS1*) are implicated in the thermotolerance of pollen tubes (Valencia-Morales et al. 2012; Yang et al. 2009); the flowering time is regulated by *AtJ3* via its direct binding to a MADS-box transcription factor (Shen et al. 2011), and *AtJ8*, *AtJ11,* and *AtJ20* are involved in the optimization of photosynthetic reactions and stabilization of photosystem II (PSII) complexes under high light stress (Chen et al. 2010). In tomato, *LeCDJ1* is also found to be essential for maintaining PSII under chilling stress in tomato (Kong et al. 2014b), and its J-domain is the key farnesylation target in meristem size control, abscisic acid signaling, and drought resistance (Barghetti et al. 2017). Lee et al. (2018) cloned the alfalfa DnaJ-like protein (*MsDJLP*) gene downstream of the strong constitutive CaMV 35S promoter and transformed it into tobacco plants, the result showed that overexpression of the *MsDJLP* gene enhances tolerance to chilling and heat stresses in transgenic tobacco plants.

Here, we identified 115 J-protein coding genes in the rice genome, and systematically analyzed the corresponding J-proteins. The classification, chromosomal localization, gene structure, domain organization, and expression profiling of J-protein genes in different tissues and under different abiotic stress conditions were performed.

## Materials and methods

### Identification of the J-protein family members in rice

Rice (*Oryza sativa*) J-proteins were indentified from the plant genomics resource Phytozome v12.1 (https://phytozome.jgj.doe.gov/pz/portal.htm1#!info?alias = Org_Osativa). First, DnaJ was used as a keyword to search for J-proteins, and all candidate proteins were then tested using the SMART database (http://smart.embl-heidelberg.de/) or the National Central for Biotechnology Information (NCBI) Batch Web CD-Search Tool (https://www.ncbi.nlm.nih.gov/Structure/bwrpsb/bwrpsb.cgi) (Zhang et al. 2018). Second, to thoroughly identify the J-proteins and avoid omission of the unannotated ones, all amino acid sequences of rice J-proteins family genes were collected and used as proteins queries to the basic local alignment search tool (BLASTp) against *A. thaliana* J-proteins. Reciprocal BLAST was used for further confirmation.

### Gene structure, domain organization, and phylogenetic analysis of rice J-protein genes

The exon–intron gene structure of J-proteins was analyzed by the online program Gene Structure Display Server GSDS 2.0 (http://gsds.cbi.pku.edu.cn/index.php) (Hu et al. 2015). The domain organizations of J-proteins family were analyzed using the SMART (http://smart.embl-heidelberg.de/), protein family (Pfam) database (http://pfam.xfam.org/), and NCBI Bath Web CD-Search (https://www.ncbi.nlm.nih.gov/cdd) databases.

The full-length amino acid sequences of rice J-protein genes were used for phylogenetic analysis. All of the acquired sequences were first aligned by Clustal X 2.0 software (Larkin et al. 2007) with the default parameters. An unrooted neighbor-joining phylogenetic tree was constructed using the MEGA6 software (Tamura et al. 2013) with bootstrap test of 1000 times. The rice J-protein genes were classified into different groups according to the topology of phylogenetic tree.

### Chromosomal localization and gene duplication

The chromosomes positions of the rice J-protein genes were acquired from the Phytozome database. The MapChart software (Voorrips 2002) was used for mapping the chromosomal positions of rice J-protein gens and to calculate their relative distances. Tandem duplications indicated that the tandemly arrayed genes with close phylogenetic relationships were located at the same chromosomal location within ~ 100 kb (Kong et al. 2007).

### Publicly available microarray data analysis

For tissue-specific expression, we used the microarray data available in the RiceXPro database (http://ricexpro.dna.affrc.go.jp/) via the accession numbers RXP_0001 (Sato et al. 2011). The normalized data were used to produce a heat map in Multi Experiment Viewer (MeV, version 4.6.0) software (Howe et al. 2010).

### Stress treatments and RNA-sequencing (RNA-seq) analysis

Rice seedlings were grown in a greenhouse at 28 °C under a 14 h day/10 h night cycle. Two-week-old seedlings were subject to heat, drought, and salt stresses following the methods of Byun (Byun et al. 2015). For the heat stress treatment, seedlings were incubated at 45 °C (Li et al. 2015). For the drought stress treatment, rice seedlings were placed into 20% polyethylene glycol 6000 (PEG-6000) solution. For the high-salinity treatment, seedlings were transferred to Murashige and Skoog (MS) medium supplemented with 200 mM NaCl and incubated at 15 °C. Total RNA was extracted from stem and leaf tissues collected at 0, 1, 3, 6, 12, and 24 h after the onset of the abiotic stress imposition. The RNA-seq data are deposited in the NCBI Sequence Read Archive (SRA, https://www.ncbi.nlm.nih.gov/Traces/study/?acc=PRJNA530826) under access number SRP190858. To obtain data suitable for cluster displays, the absolute number of fragments per kilobase of transcript per million mapped reads (FPKM) was divided by the mean of all FPKM values, and the ratios were log 2 transformed. Multi Experiment Viewer v. 4.6.0 (Howe et al. 2010) was used to generate the heat map.

## Results and discussion

### Identification and analysis of rice J-protein genes

A previous study reported that 104 J-protein genes in rice (Sarka et al. 2013). Here, we examined the published data and rescreened rice J-protein gene family members in the Phytozome database (Supplementary Table 1). We obtained 115 J-protein genes in rice and indentified 11 novel genes (such as Os12g31460, Os08g03380, Os10g33790, Os01g70250, Os07g32950, Os07g43870, Os07g42800, Os03g27460, Os12g44260, Os03g19200, and Os07g49000). The phylogenetic relationships among J-protein genes provided a new perspective for the classification of J-proteins, and the molecular weights of J-proteins ranged between 10.20 kDa (Os12g36180) and 287.69 kDa (Os10g42439).

### Gene structure, domain organization, and phylogenetic analysis of rice J-proteins

**Fig. 1:**
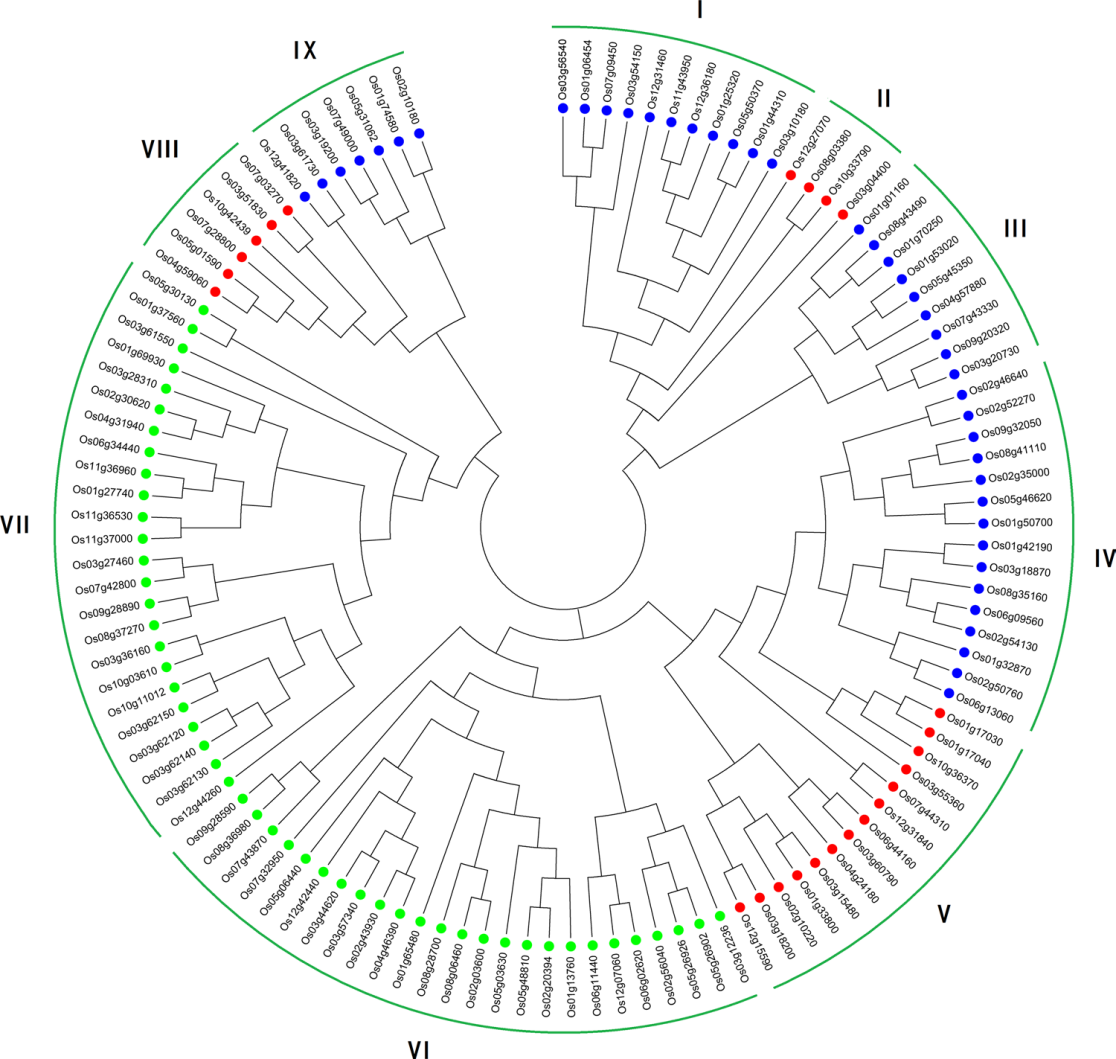
Phylogenetic analysis of rice J-proteins. Full-length amino acid sequences of 115 rice J-proteins were aligned via Clustal X, and the phylogenetic tree was constructed in MEGA6 using the neighbor-joining method with 1000 bootstrap replicates. The oligo-gene clades (I, III, IV, and IX) are indicated by blue dots, mono-gene clades (II, V, and VIII) are indicated by red dots, and multigene clades (VI and VII) are indicated by green dots

To analyze the evolutionary relationships among J-proteins in rice, 115 amino acid sequences were used to construct a phylogenetic tree (Fig. 1). Recently, studies have reported that the J-proteins of *A. thaliana and Brassica oleracea* are divided into 15 major clades, which contain more than ten members (multigene clades), two-to-seven members (oligo-gene clades), or a single member (mono-gene clades) (Zhang et al. 2018). Based on the phylogenetic relationships obtained here, rice J-proteins are divided into nine clades (I–IX). Gene organization plays a vital role in the evolution of multiple gene families (Xu et al. 2012). The percentage of intronless J-protein genes obtained here (20.00%) was similar to that of *A. thaliana* (22.22%, Zhang et al. 2018) and *B. oleracea* (23.26%, Zhang et al. 2018). The correlation between intron numbers and J-domain numbers further confirmed the classification of rice J-proteins. In the previous studies, genes with few or no introns were considered to have enhanced expression levels in plants (Chung et al. 2006; Ren et al. 2006). To response timely to various stresses, genes must be rapidly activated, which would be assisted by a compact gene structure with less introns (Jeffares et al. 2008).

**Fig. 2:**
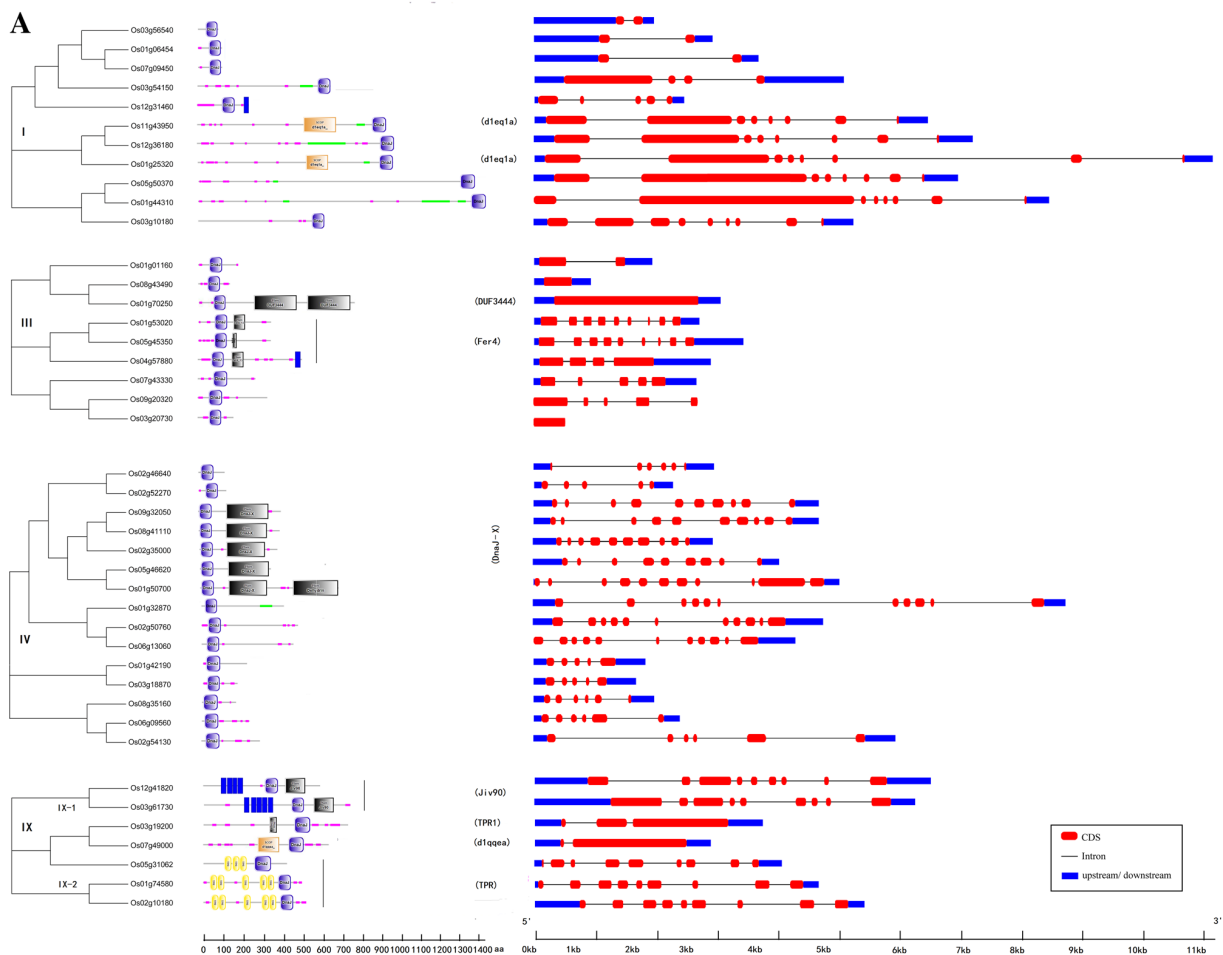

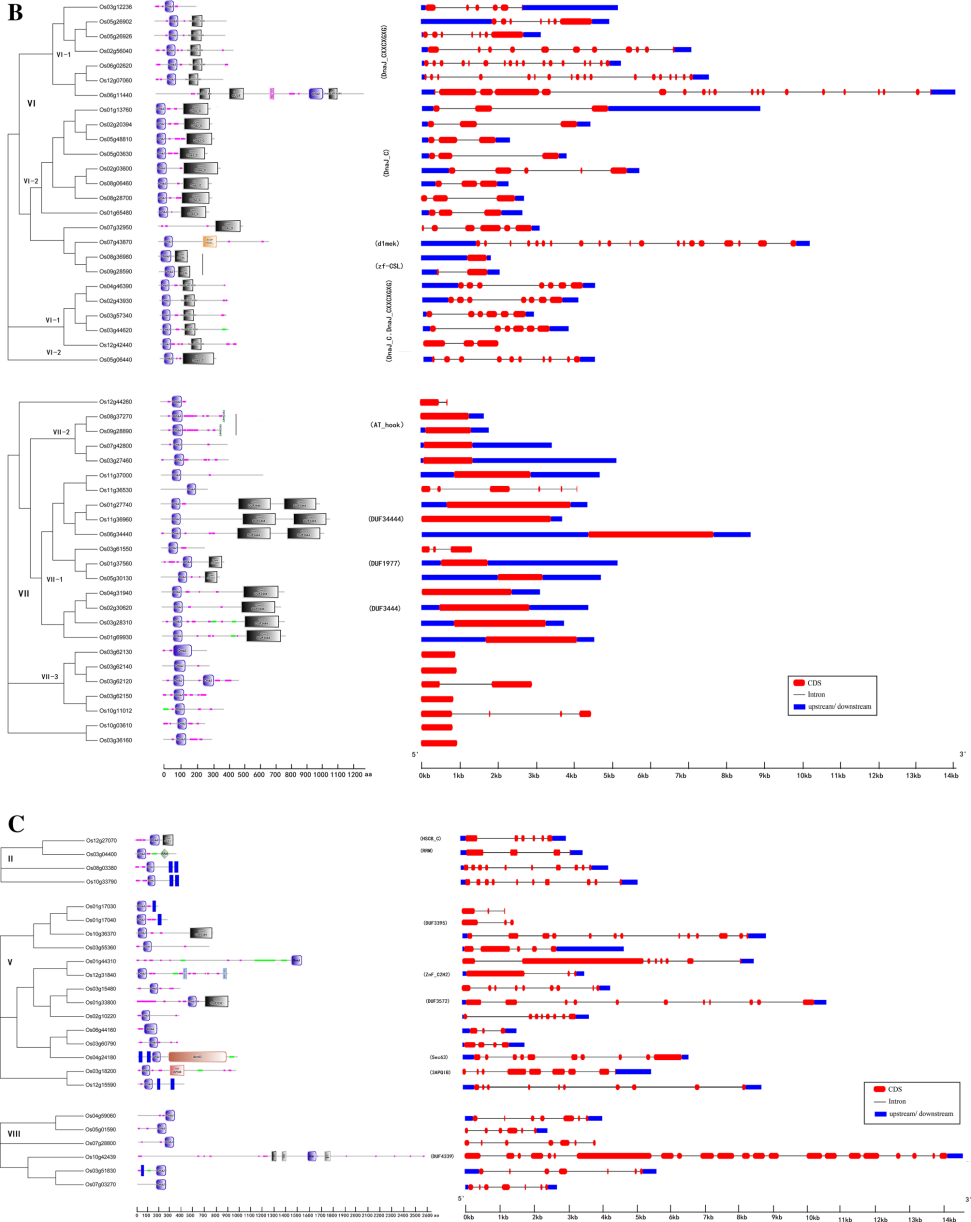
Phylogenetic analysis, domain architecture, and gene structure in the three types of clades. The unrooted neighbor-joining (NJ) tree was generated in MEGA6 with parameter settings, as stated in Fig. 1, and based on full-length amino acid sequences of J-proteins in rice. The red solid boxes represent exons, blue represents genes up/down stream, and black lines represent introns. **a** Oligo-gene clades. **b** Multigene clades. **c** Mono-gene clades

Oligo-gene clades in rice included clades I, III, IV, and IX (Fig. 2a). The genes in clade I comprised multiple introns, except for Os03g56540, Os01g06454, and Os07g09450 genes that contained a single intron. Members of this clade contained only J-domain at the C-terminus, but Os11g43950 and Os01g25320 genes also contained the d1eq1a domain before the J-domain. In clade III genes, all J-domains were located at the central region, and Os01g53020 and Os04g57880 also contained a Fer4_13 domain after the J-domain. The Fer4_13 domain was first identified in a sulfate-reducing bacterium (Sery et al. 1994), which contained a ferredoxin domain [4Fe–4S] cluster (Dorn et al. 2010), likely acquired through horizontal gene transfer events (Petitjean et al. 2012). The genes in clade IV comprised 4–10 introns, and all the J-domains of this clade were located at the N-terminus. Genes Os09g32050, Os08g41110, Os02g35000, Os05g46620, and Os01g50700 also contained a DnaJ-X with unknown function. Clade IX genes comprised eight introns, except Os03g19200 and Os07g49000 genes, and this clade was divided into two subclades, IX-1 and IX-2. The J-domains of IX-1 genes were located between a transmembrane domain (TMD) and the Jiv90 domain, indicating the region, where the bovine J-protein Jiv interacts with viral polyproteins (Muller et al. 2003). The J-domains of IX-2 proteins were located at the C-terminus, and there were multiple tandem tetratricopeptide repeat (TPR) domains before the J-domain. TPR domain is a structural motif of present in a wide range of proteins, and it mediates protein–protein interactions (Muller et al. 2003) and can couple with various domains to perform diverse functions (Prasad et al. 2010).

Multigene clades of rice included clades VI and VII (Fig. 2b). Genes within clade VI usually contained the second distinctive DnaJ C-terminal domain DnaJ_C, but this was absent in Os03g12236, Os07g43870, Os08g36980, and Os09g28590. Clade VI was divided into two subclades, VI-1 and VI-2, containing 12 and 13 members, respectively. The genes in subclade VI-1 usually had more introns than those in subclade VI-2, except Os07g43870 and Os05g06440. Subclade VI-1 genes usually had the DnaJ_CXXCXGXG domain, which contained four cysteine-rich repeats of the motif CXXCXGXG and was imbedded in the N-terminus of DnaJ_C domain. Genes Os07g43870 and Os05g06440 displayed the zf-CSL domain instead, which contained four conserved cysteine residues to chelate a single zinc ion (Sun et al. 2005). The genes in clade VII lacked introns or had few introns. Clade VII was divided into three subclades, VII-1, VII-2, and VII-3, and most members in subclade VII-1 had a single DUF1977 domain, or a single or double C-terminal DUF3444 domain with unknown function. However, Os08g37270 and Os09g28890 in subclade VII-2 displayed a C-terminal DNA-binding domain with preference for A/T-rich regions (AT-hook), which is found in mammalian HMGI/Y proteins (Reeves and Beckerbauer 2001). Subclade VII-3 genes contained a single J-domain and lacked the C-terminal AT-hook, DUF1977, and DUF3444 domains, except Os03g62120.

The mono-gene clades corresponded to small, disperse branches with distant relationships among them and separated by well-supported clades. In the mono-gene clades of rice included clades II, V, and VIII (Fig. 2c), and almost every rice J-protein gene contained multiple introns and represented an individual clade. Genes with closer relationships usually displayed similar gene structure and protein domain organization; here, we will focus on some particular genes. The Os12g27070 gene in Clade II contained the C-terminal oligomerization domain HSCB_C found in heat shock cognate protein B (Ciesielski et al. 2012), while Os03g04400 contained the C-terminal recognition motif RRM, which is found in RNA and DNA-binding proteins (Birney et al. 1993). Genes Os08g03380 and Os10g33790 contained double C-terminal TMD domains. The Os12g31840 gene in Clade V contained a double zinc-finger (ZnF_C2HC) domain at the central region and C-terminus, while Os10g36370 and Os01g33800 genes contained the C-terminal DUF3395 and DUF3752 domains, respectively, with unknown functions. The Os04g24180 gene contained a pair of TMD domains at the N-terminus and a Sec63 domain at the C-terminus. The Sec63 domain was named after the yeast Sec63p, and it is involved in the biogenesis of secretory and transmembrane proteins (Servas and Romisch 2013). Genes Os01g17030 and Os01g17040 encompassed a C-terminal TMD domain, and gene Os12g15590 contained a pair of TMD domains at the C-terminus. The Os10g42439 gene in clade VIII contained one DUF4339 domain, a typical J-protein domain, and two armadillo (ARM) domains at the central region. The DUF4339 domain is functionally uncharacterized, and the ARM domain, which is a tandemly repeated sequence motif, might be involved in transducing the Wingless/Wnt signal (Hatzfeld 1999).

### Chromosomal location of J-protein genes in rice

**Fig. 3:**
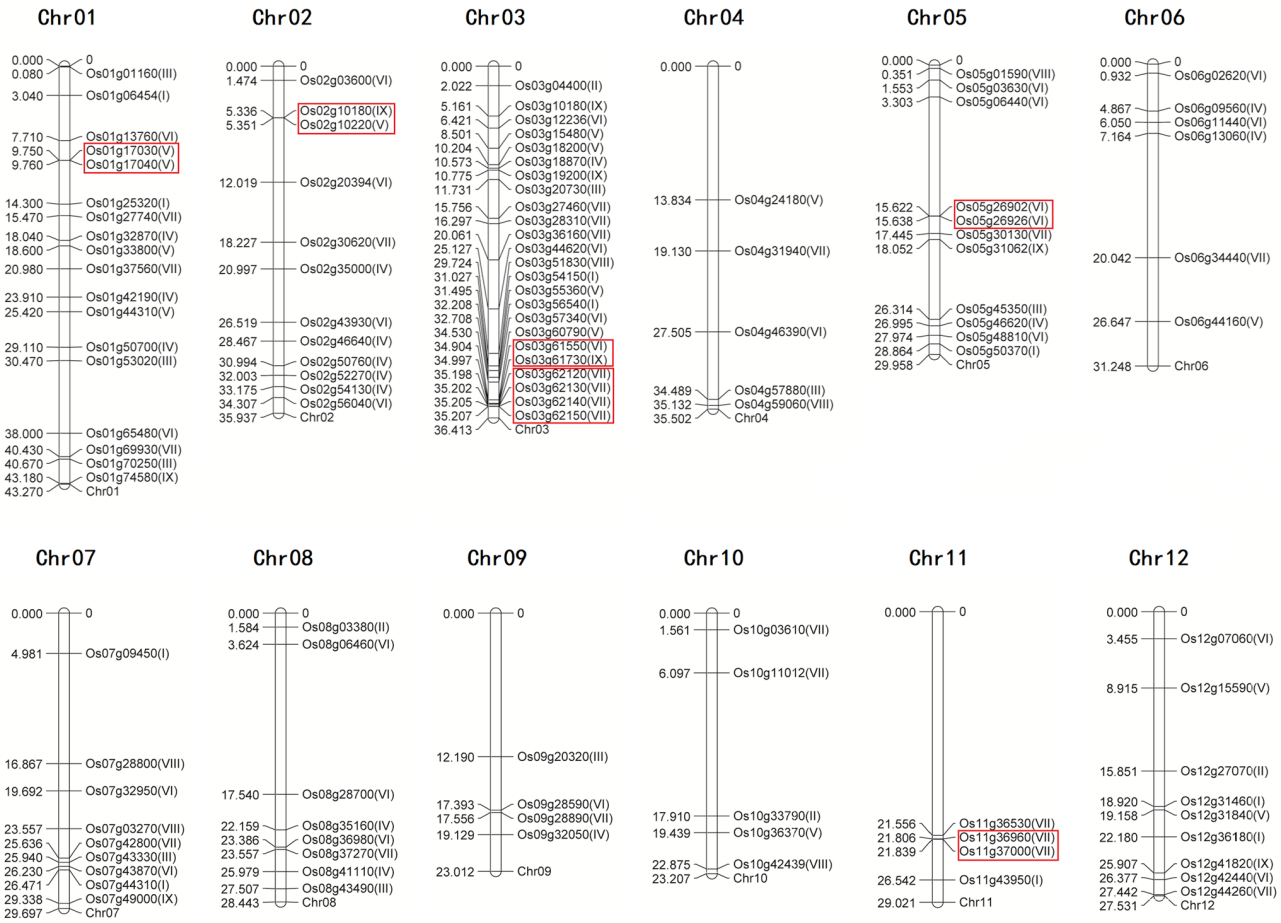
Chromosomal distribution of J-protein genes in rice. Tandemly duplicated genes are indicated by the red box. Values on the left of each chromosome represent megabases (Mb) and the chromosome number is indicated at the top of each chromosome. Roman numerals in parentheses indicate the corresponding gene clades obtained in the present study

The 115 rice J-protein family genes were randomly distributed on all chromosomes (Fig. 3). Chromosomal distribution of genes in each clade was usually uneven. The maximum number of 24 genes (20.87%) was present on chromosome (chr) 3, and only four genes (3.48%) were found on chr 9 and chr 11. Clade VII genes were distributed on all chromosomes, clade VI had five genes on chr 5, but no genes on chromosomes 10 and 11. In addition, five separate pairs of tandem duplicated genes were located on chromosomes 1, 2, 3, 5, and 11. Four tandem duplicated genes were located on chr 3.

### Expression patterns of rice J-protein genes in different tissues

**Fig. 4:**
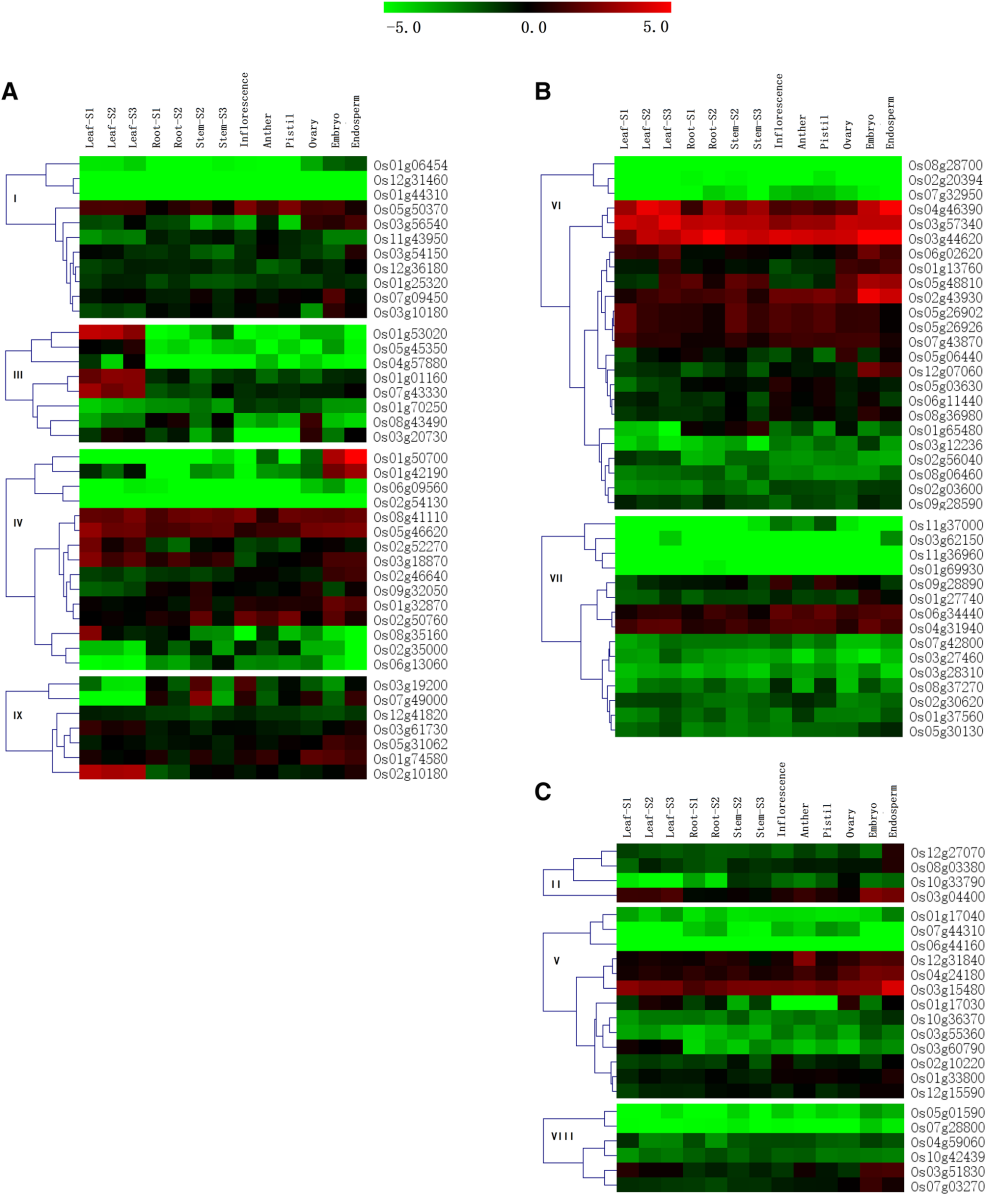
Expression profiles of rice J-protein family genes in various tissues. Microarray data were used to produce the heat maps, Red and green indicate high and low expression levels in the 13 tissues. S1 vegetative stage, S2 reproductive stage, S3 ripening stage. **a** Expression of Oligo-gene clade members, **b** expression of multigene clade members, **c** expression of mono-gene clade members

The different rice J-protein genes showed distinct expression patterns (Fig. 4). Genes Os08g41110, Os05g46620, Os04g46390, Os03g57340, Os03g44620, Os02g43930, Os04g31940, and Os03g15480 were constitutively expressed at a high level in nearly all tissues and organs, and most of them contained other domains besides the J-domain. Forty other genes were also expressed constitutively, but at low level. Genes Os01g53020, Os01g01160, Os07g43330, Os02g52270, and Os02g10180 showed relatively higher expression levels only in leaf. Genes Os01g50700, Os01g42190, Os02g46640, and Os12g07060 had slightly higher expression levels in the embryo and endosperm than in other tissues. Similar to several J-protein genes in pepper, eight genes showed specific housekeeping expression activity (Fan et al. 2017). Moreover, the expression profile showed that the 61 rice J-protein genes were expressed in at least one tissue. The result implied that they could be involved in the process of rice growth and development.

### Differential expression of rice J-protein genes under abiotic stresses

**Fig. 5:**
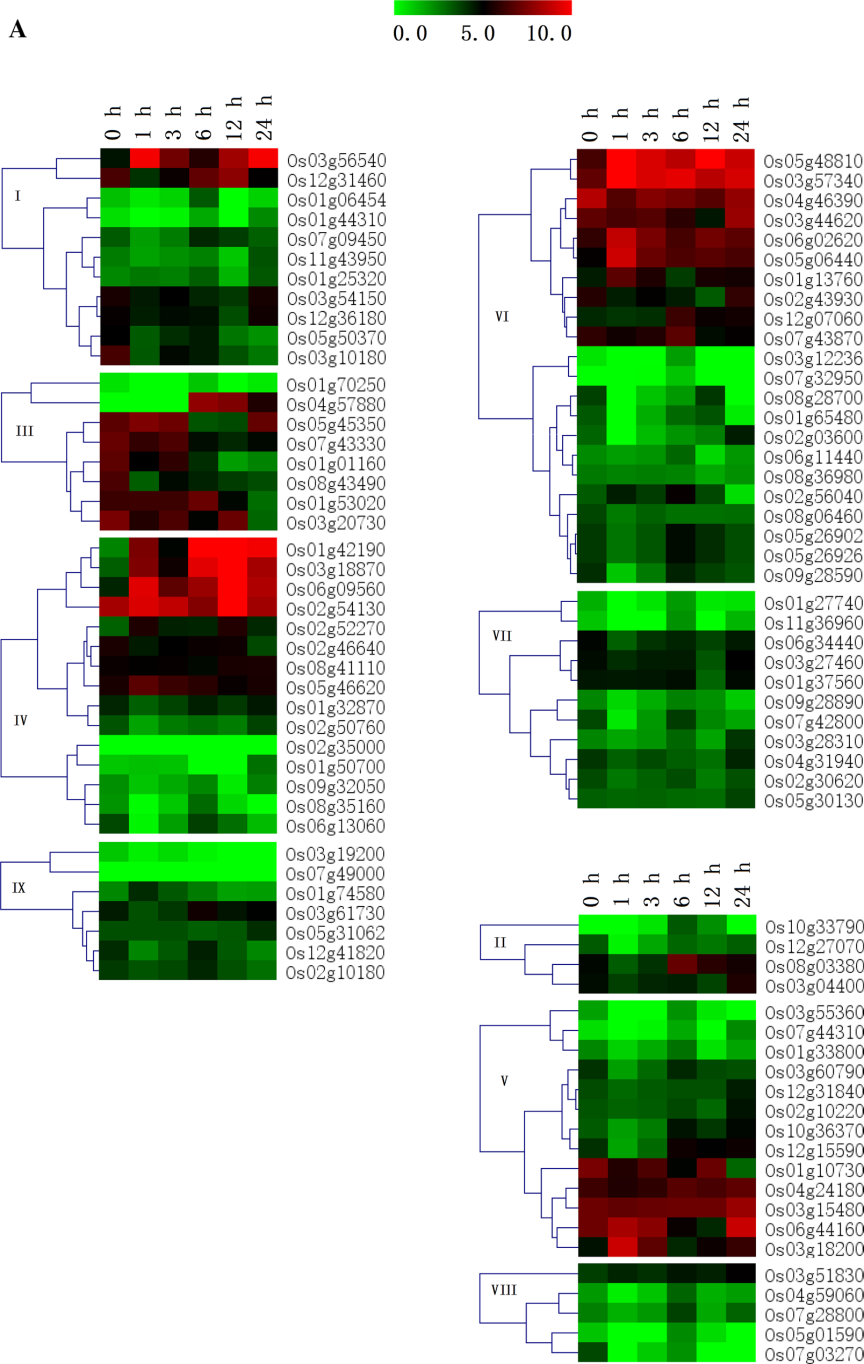

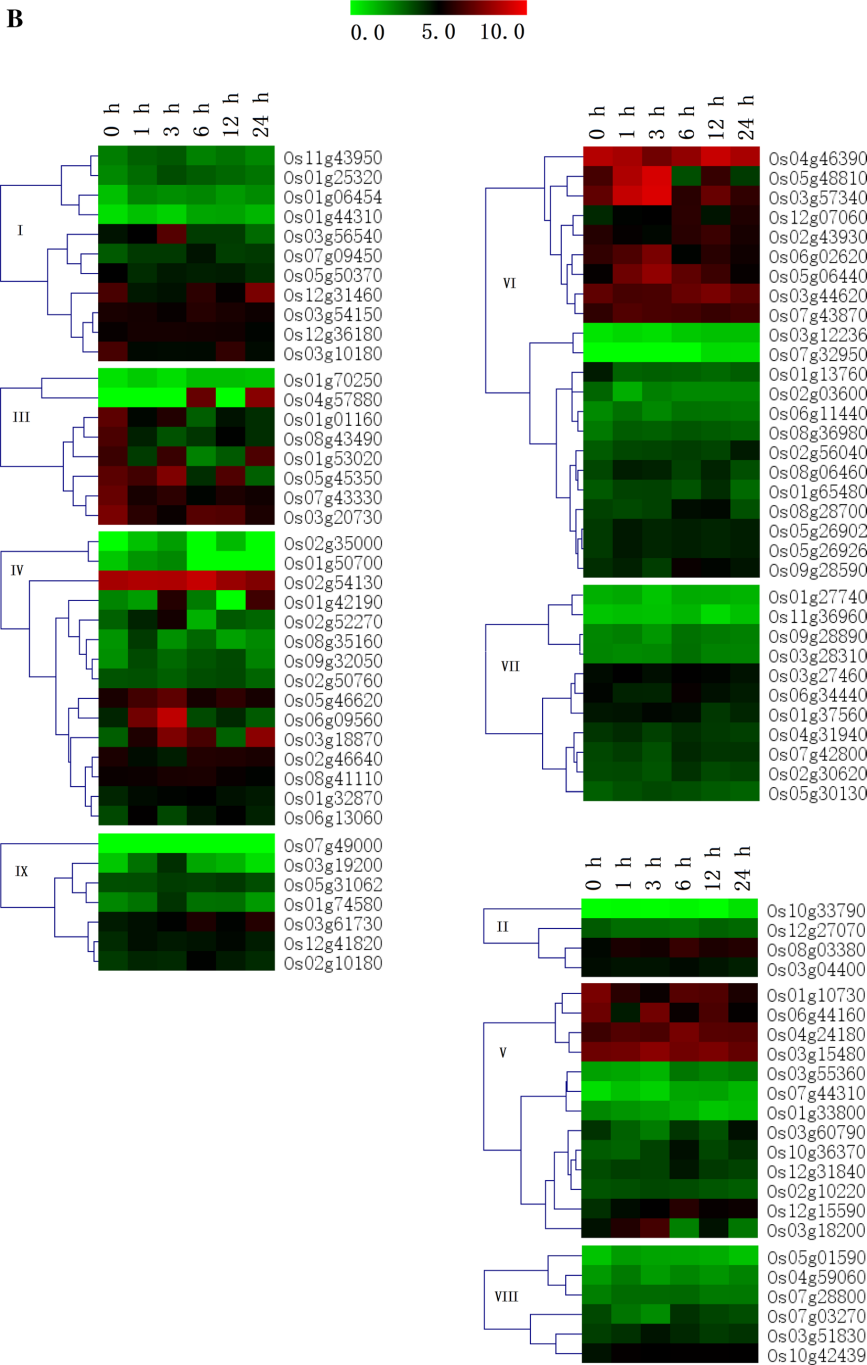

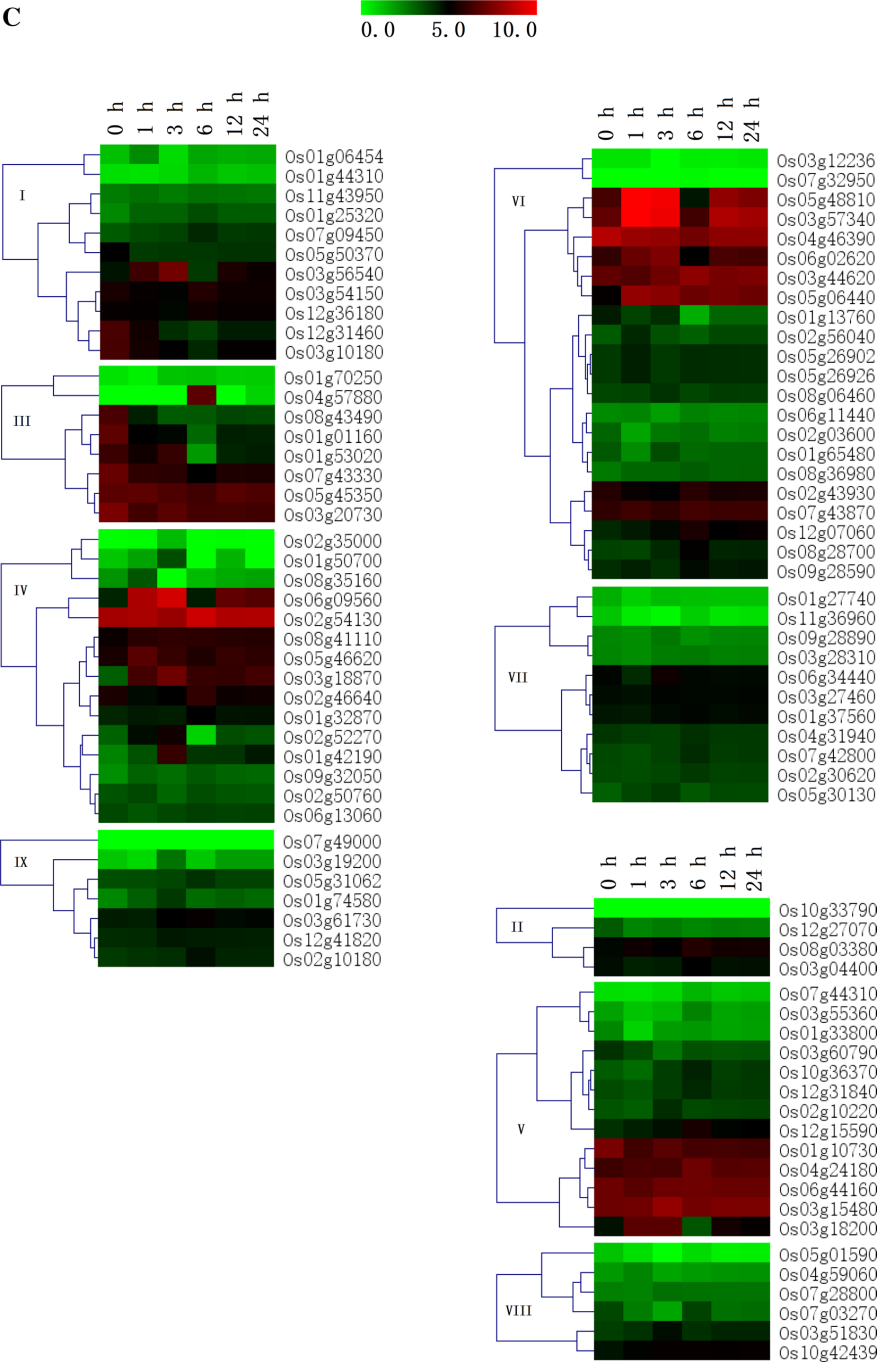
Expression profiles of rice J-protein genes under abiotic stresses. RNA-seq data were used to produce the heat maps. Red and green indicate high and low expression levels, respectively. **a** Expression profiles of rice J-protein family genes under heat stress. **b** Expression profiles of rice J-protein family genes under drought stress. **c** Expression profiles of rice J-protein family genes under salt stresses

Based on the RNA-seq data, three heat maps of rice J-protein genes, representing the FPKM values under heat, drought (PEG), and salt stress treatments were obtained (Fig. 5). Under heat stress (Fig. 5a), most of J-protein genes were up-regulated at 6 h. Genes Os03g56540, Os05g45350, Os02g54130, Os06g09560, Os03g18200, Os03g57340, Os01g13760, Os05g48810, Os06g02620, Os05g06440, and Os01g74580 were highly expressed at 1 h, but down-regulated at 3 h. Some genes, such as Os03g44620, Os12g31840, Os02g03600, Os03g28310, Os03g56540, Os01g42190, Os06g44160, Os03g15480, Os03g51830, Os03g04400, and Os01g50700, were highly expressed at 24 h. In addition, most of the genes in clades VII, VIII, and IX showed lower transcription levels at each time point under heat stress than under control conditions. Some studies have shown that *AtDjA2* and *AtDjA3* function in the improvement of *A. thaliana* thermotolerance (Li et al. 2007) and that *TMS1* plays an important role in the thermotolerance of pollen tubes (Yang et al. 2009). While *AtDjB1* plays a crucial role in maintaining redox homeostasis, and facilitates thermotolerance by protecting cells against heat-induced oxidative damage (Zhou et al. 2012). *LeCDJ1* overexpression enhanced tolerance to heat stress in transgenic tomato (Kong et al. 2014a). Overexpression of *SlCDJ2* in tomato also facilitated thermotolerance by protecting ribulose-1,5-bisphosphate carboxylase/oxygenase (Rubisco) activity and maintaining carbon assimilation capacity in response to heat stress (Wang et al. 2015). *SlDnaJ20* overexpression enhances the thermotolerance of transgenic tomatoes, whereas the suppression of *SlDnaJ20* increases the heat sensitivity of transgenic tomatoes (Wang et al. 2019).

Under drought stress (Fig. 5b), most of the J-protein genes showed elevated transcription levels at 3 and 6 h, and the expression of some J-protein genes reached their peak at 3 h, particularly Os03g56540, Os05g45350, Os05g46620, Os06g09560, Os05g48810, Os03g57340, Os06g02620, Os05g06440, Os06g44160, Os03g15480, and Os03g18200. Only a few J-protein genes showed an increased expression at 24 h, such as Os12g31460, Os01g53020, Os04g57880, Os01g42190, and Os03g18870. Genes Os01g44310, Os01g70250, Os02g35000, Os07g49000, Os03g12236, Os07g32950, Os01g27740, Os11g36960, Os10g33790, Os07g44310, and Os05g01590 always showed lower transcription levels under drought stress than under control conditions. Previous studies showed that the overexpression of *NtDnaJ1* in *A. thaliana* plants enhanced their tolerance to osmotic or drought stress (Xia et al. 2014) and that Hsps40, encoded by the *J2* and *J3* genes, conferred abscisic acid hypersensitivity and drought resistance (Barghetti et al. 2017). Overexpression of a tomato chloroplast-targeted DnaJ gene enhanced the tolerance to drought stress and the resistance to *Pseudomonas solanacearum* of transgenic tobacco (Wang et al. 2014).

When the plants were subjected to the salt stress (Fig. 5c), the expression levels of most J-protein genes changed only slightly, but Os03g56540, Os04g57880, Os06g09560, Os05g46620, Os03g18870, Os02g52270, Os01g42190, Os05g48810, Os03g57340, Os06g02620, and Os05g06440 were obviously up-regulated at 3 h or 6 h. Interestingly, the ten genes mentioned above that showed lower transcription levels under drought stress than under control conditions at all timepoints, also maintained lower transcription levels at each timepoint under salt stress. It has been reported that *ANJl* can complement the yeast *mas5* temperature-sensitive mutation, and its expression is induced by heat shock and salt stress (Zhu et al. 1993). Overexpressed DnaJ in transgenic *A. thaliana* plants showed increased NaCl tolerance compared with the wild-type genotype (Zhao et al. 2010), and *AtDjA3* null mutant shows increased sensitivity to salt stress in germination and post-germination stages (Salas-Muñoz et al. 2016).

### J-proteins are involved in the molecular mechanism of Hsp70 and their regulatory networks during plant development or environmental stresses

**Fig. 6:**
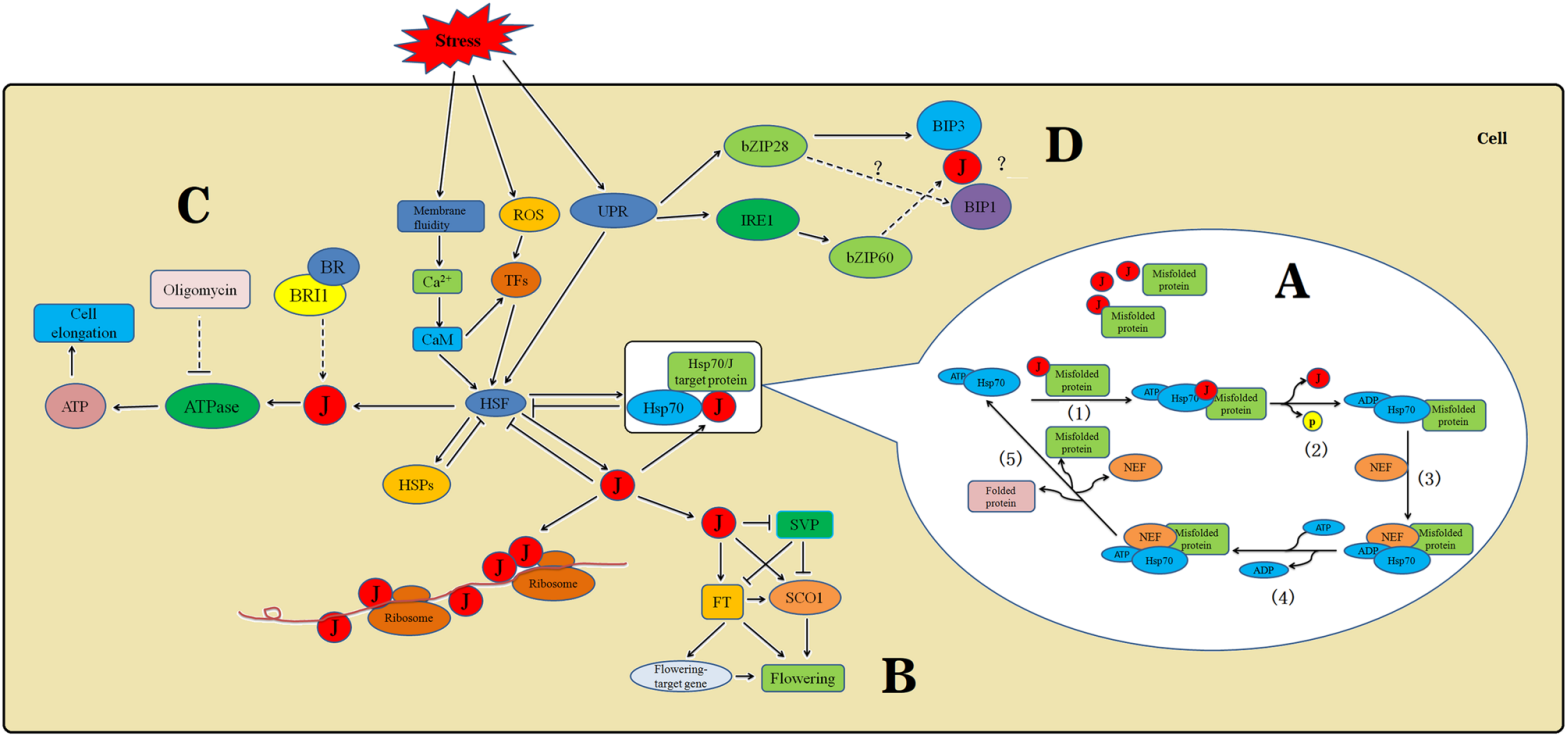
Proposed model integrating J-proteins as key molecular chaperones involve in regulatory networks during plant development or environmental stresses. **a** Hsp70 machinery reaction cycle. **b** *J3* regulates flowering time by mediating *SVP* activity to regulate *SOC1* and *FT* transcription. **c** *BIL2* induces cell elongation through BR signaling to promote ATP synthesis in the mitochondria. **d** Possible pathways initiated by ER stress that can lead to cell survival or cell death. J J-protein, *ROS* reactive oxygen species, *Cam* Ca^2+^-calmodulin, *TFS* transcription factors, *HSF* heat shock factor, *HSP* heat shock protein, *UPR* unfolded protein response, *BR* brassinosteroid, *BRI1* brassinosteroid insensitive 1, *ATP* adenosine triphosphate, *ADP* adenosine diphosphate, *bZIP* basic leucine zipper protein, *BIP* binding immunoglobulin proteins, *IRE1* inositol-requiring enzyme 1, *SVP* short vegetative phase, *FT* flowering locus T, *SCO1* suppressor of overexpression of constans 1, *NEF* nucleotide exchange factor, arrows represent positive regulation, bars indicate negative regulation, broken arrows indicate possible but not firmly demonstrated routes, and (?) indicates unknown steps

J-proteins, as key molecular chaperones, not only respond to abiotic stresses, but are also involved in the molecular mechanism of Hsp70 and their regulatory networks during plant development or environmental stresses (Fig. 6). It was reported that J-proteins can bind to abnormally folded substrate proteins via the zinc finger or C-terminal domains, and transfer substrate proteins to HSP70-ATP by interacting with Hsp70 (Fig. 6a), and this process can be accomplished via five steps (Shiber and Ravid 2014). The *A. thaliana DNAJ HOMOLOG 3* (*J3*), which mediates the integration of flowering signals through its interaction with *short vegetative phase* (*SVP*) (Fig. 6b), which acts as a key flowering regulator that represses the expression of *flowering locus t* (*ft*) and *suppressor of overexpression of constans 1* (*SOC1*). Thus, *J3* promotes flowering partly through upregulating the expression of *SOC1* and *FT* (Shen et al. 2011). Bekhochir et al. (2013) used Brassinazole (Brz)-mediated chemical genetics to identify *Brz*-*insensitive*-*long hypocotyls 2*-*1D* (*bil2*-*1D*), and the *BIL2* gene encodes a mitochondrial-localized J-protein family that is involved in protein folding (Fig. 6c). In addition, *BIL2* acts downstream of the brassinosteroid (BR) receptor brassinosteroid insensitive 1 (BRI1) and induces cell elongation by promoting ATP synthesis in mitochondria, and participates in resistance against salt and strong light stresses. The previous studies showed that *A. thaliana TMS1* encodes Hsp identical to the J-protein AtERdj3A and plays important roles in the thermotolerance of pollen tubes and other plant tissues (Howell 2013; Zhao et al. 2015). In response to ER stress, two mechanisms can be initiated (Fig. 6d), one arm involves membrane-associated transcription factors such as bZIP28, and the other involves the membrane-associated dual-functioning protein kinase/ribonuclease called inositol-requiring enzyme 1 (IRE1), which splices the mRNA encoding bZIP60 (Howell 2013). Both bZIP28 and IRE1 are activated by the accumulation of misfolded proteins in the ER. The bZIP28 is mobilized from the ER and transported to Golgi bodies, and relocated to the nucleus after cleavage releasing its N-terminal component of bZIP28 into the cytosol. Once activated, IRE1 splices the bZIP60-encoding mRNA, creating a frame shift that induces the spliced RNA to encode a transcription factor with a nuclear targeting signal. Both bZIP28 and bZIP60 can heterodimerize, and the two mechanisms of this signaling pathway may converge in the formation of heterodimers that can up-regulate stress response genes. The J-domain of *TMS1* might interact with binding immunoglobulin proteins 1 (BiP1) and binding immunoglobulin proteins 3 (BiP3), and stimulate their ATPase enzyme activities, leading to the degradation of unfolded and misfolded proteins (Zhao et al. 2015). In addition, *TMS1* may function downstream of bZIP28 and bZIP60 and thus be involved in plants’ thermotolerance.

## Conclusion

In summary, 115 putative rice J-protein genes were identified and classified into nine major clades (I–IX), according to their phylogenetic relationships. These J-protein genes were randomly distributed on 12 chromosomes. Gene-structure analysis revealed that most J-protein genes of clade VII were intronless. Expression profile showed that the 61 rice J-protein genes were expressed in at least one tissue. The result implied that they could be involved in the process of rice growth and development. The RNA-seq data demonstrated that 96 genes were differentially expressed under heat, drought, and salt stresses; 57 genes were up-regulated and 39 were down-regulated under heat stress, 65 genes were up-regulated and 31 were down-regulated under drought stress, and 60 genes were up-regulated and 36 were down-regulated under salt stress at 6 h. These results indicate that J-proteins might have important roles in response to abiotic stresses.

## Electronic supplementary material

Below is the link to the electronic supplementary material.

Supplementary material 1 (DOCX 68 kb). List of J-protein genes family in rice. Chr chromosome, AA number of amino acid, MW Molecular weight KDa, PI theoretical isoelectric point, DnaJ_CXXCXGXG zinc-finger domain, DnaJ_C C-terminal domain, DUF3444, DUF1997, DUF4339, DUF3395 and DUF3752 domain of function unknown, d1eq1a, d1mek and d1qqea domain of function unknown; HSCB_C C-terminal oligomerisation domain (heat shock cognate protein B), RRM RNA recognition motif, Fre4 ferredoxin domain [4Fe–4S] cluster, DnaJ-X X lies between the N-terminal DnaJ and the C-terminal Z domains, Sec63 named after the yeast Sec63, 3APQlB domain of function unknown, zf-CSL small protein motifs which contain multiple finger-like protrusions, AT_hook DNA-binding motifs with a preference for A/T rich regions, Jiv90 the region of a bovine J-protein Jiv interacting with viral polyprotein, TPR tetratricopeptide region, Up Up-regulation, Down Down-regulation, / represents the genes have no corresponding probe sets in the microarray data. *represents novel members of rice J-proteins

## References

[CR1] Al-Whaibi MH (2011). Plant heat-shock proteins: a mini review. King Saud Univ Sci.

[CR2] Barghetti A, Sjögren L, Floris M, Paredes EB, Wenkel S, Brodersen P (2017). Heat-shock protein 40 is the key farnesylation target in meristem size control, abscisic acid signaling, and drought resistance. Gene Dev.

[CR3] Bekhochir D, Shimada S, Yamagami A, Kanda S, Oganwa K, Nakazawa M, Matsui M, Sakuta M, Osada H, Asami T, Nakano T (2013). A novel mitochondrial DnaJ/Hsp40 family protein BIL2 promotes plant growth and resistance against environmental stress in brassinosteroid signaling. Planta.

[CR4] Birney E, Kumar S, Krainer AR (1993). Analysis of the RNA-recognition motif and RS and RGG domains: conservation in metazoan pre-mRNA splicing factors. Nucleic Acids Res.

[CR5] Byun MY, Lee J, Cui LH, Kang Y, Oh TK, Park H, Lee H, Kim WT (2015). Constitutive expression of *DaCBF7*, an Antarctic vascular plant *Deschampsia antarctica* CBF homolog, resulted in improved cold tolerance in transgenic rice plants. Plant Sci.

[CR6] Chen KM, Holmström M, Raksajit W, Suorsa M, Piippo M, Aro EM (2010). Small chloroplast-targeted DnaJ proteins are involved in optimization of photosynthetic reactions in *Arabidopsis thaliana*. BMC Plant Biol.

[CR7] Chiu CC, Chen LJ, Su PH, Li HM (2013). Evolution of chloroplast J proteins. PLoS One.

[CR8] Chung BY, Simons C, Firth AE, Brown CM, Hellens RP (2006). Effect of 5′UTR introns on gene expression in *Arabidopsis thaliana*. BMC Genom.

[CR9] Ciesielski SJ, Schilke BA, Osipiuk J, Bigelow L, Mulligan R, Majewska J, Joachimiak A, Marszalek J, Craig EA, Dutkiewicz R (2012). Interaction of J-protein co-chaperone Jac1 with Fe–S scaffold Isu is indispensable in vivo and conserved in evolution. J Mol Biol.

[CR10] Craig EA, Marszalek J (2017). How do J-proteins get Hsp70 to do so many different things?. Trends Biochem Sci.

[CR11] De CA, Kohiyama M, Richarme G (1995). A novel function of *Escherichia coli* chaperone DnaJ Protein-disulfide isomerase. J Biol Chem.

[CR12] Dorn KV, Willmund F, Schwarz C, Henselmann C, Pohl T, Hess B, Veyel D, Usadel B, Friedrich T, Nickelsen J, Schroda M (2010). Chloroplast DnaJ-like proteins 3 and 4 (CDJ3/4) from *Chlamydomonas reinhardtii* contain redox-active Fe–S clusters and interact with stromal HSP70B. Biochem J.

[CR13] Fan FF, Yang X, Cheng Y, Kang Y, Chai X (2017). The DnaJ gene family in pepper (*Capsicum annuum L.*): comprehensive identification, characterization and expression profiles. Front Plant Sci.

[CR14] Georgopoulos C, Welch WJ (1993). Role of the major heat shock proteins as molecular chaperones. Annu Rev Cell Biol.

[CR15] Georgopoulos CP, Lundquist-Heil A, Yochem J, Feiss M (1980). Identification of the *E. coli dnaJ* gene product. Mol Gen Genet.

[CR16] Gupta SC, Sharma AA, Mishra M, Mishra RK, Chowdhuri DK (2010). Heat shock proteins in toxicology: how close and how far?. Life Sci.

[CR17] Hatzfeld M (1999). The armadillo family of structural proteins. Int Rev Cytol.

[CR18] Howe E, Holton K, Nair S, Schlauch D, Sinha R, Quackenbush J (2010). MeV: multiexperiment viewer. In: biomedical informatics for cancer research.

[CR19] Howell SH (2013). Endoplasmic reticulum stress responses in plants. Annu Rev Plant Biol.

[CR20] Hu B, Jin J, Guo AY, Zhang H, Luo J, Gao G (2015). GSDS 2.0: an upgraded gene feature visualization server. Bioinformatics.

[CR21] Jeffares DC, Penkett CJ, Bähler J (2008). Rapidly regulated genes are intron poor. Trends Genet.

[CR22] Kampinga HH, Craig EA (2010). The HSP70 chaperone machinery: j proteins as drivers of functional specificity. Nat Rev Mol Cell Biol.

[CR23] Kong HZ, Landherr HLL, Frohlich MW, Leebens-Mack J, Ma H, dePamphilis CW (2007). Patterns of gene duplication in the plant *SKP1* gene family in angiosperms: evidence for multiple mechanisms of rapid gene birth. Plant J.

[CR24] Kong FY, Deng YS, Wang GD, Wang JR, Liang XQ, Meng QW (2014). LeCDJ1, a chloroplast DnaJ protein, facilitates heat tolerance in transgenic tomatoes. J Integr Plant Biol.

[CR25] Kong FY, Deng YS, Zhou B, Wang GD, Wang Y, Meng QW (2014). A chloroplast-targeted DnaJ protein contributes to maintenance of photosystem II under chilling stress. J Exp Bot.

[CR26] Kosová K, Vítámvás P, Prášil IT, Renaut J (2011). Plant proteome changes under abiotic stress–contribution of proteomics studies to understanding plant stress response. J Proteomics.

[CR27] Kurepa J, Wang SH, Li Y, Zaitlin D, Pierce AJ, Smalle JA (2009). Loss of 26S proteasome function leads to increased cell size and decreased cell number in *Arabidopsis* shoot organs. Plant Physiol.

[CR28] Larkin M, Blackshields G, Brown NP, Chenna R, McGettigan PA, McWilliam H (2007). Clustal W and Clustal X version 2.0. Bioinformatics.

[CR29] Lee KW, Rahman MA, Kim KY, Choi GJ, Cha JY, Cheong MS, Shohael AM, Jones C, Lee SH (2018). Overexpression of the alfalfa DnaJ-like protein (*Ms*DJLP) gene enhances tolerance to chilling and heat stresses in transgenic tobacco plants. Turk J Biol.

[CR30] Li GL, Chang H, Li B, Zhou W, Sun DY, Zhou RG (2007). The roles of the atDjA2 and atDjA3 molecular chaperone proteins in improving thermotolerance of *Arabidopsis thaliana* seedlings. Plant Sci.

[CR31] Li XM, Chao DY, Wu Y, Huang XH, Chen K, Cui LG, Su L, Ye WW, Chen H, Chen HC, Dong NQ, Guo T, Shi M, Feng Q, Zhang P, Han B, Shan JX, Gao JP, Lin HX (2015). Natural alleles of a proteasome α2 subunit gene contribute to thermotolerance and adaptation of African rice. Nat Genet.

[CR32] Lindquist S, Craig EA (1988). The heat-shock proteins. Annu Rev Genet.

[CR33] Miernyk JA (2001). The J-domain proteins of *Arabidopsis thaliana*: an unexpectedly large and diverse family of chaperones. Cell Stress Chaperon.

[CR34] Miot M, Reidy M, Doyle SM, Hoskins JR, Johnston DM, Genest O, Vitery MC, Masison DC, Wickner S (2011). Species-specific collaboration of heat shock proteins (Hsp) 70 and 100 in thermotolerance and protein disaggregation. Proc Natl Acad Sci.

[CR35] Muller A, Rinck G, Thiel HJ, Tautz N (2003). Cell-derived sequences in the N-terminal region of the polyprotein of a cytopathogenic pestivirus. J Virol.

[CR36] Ohta M, Wakasa Y, Takahashi H, Hayashi S, Kudo K, Takaiwa F (2013). Analysis of rice ER-resident J-proteins reveals diversity and functional differentiation of the ER-resident Hsp70 system in plants. J Exp Bot.

[CR37] Petitjean C, Moreira D, Lopez-Garcia P, Brochier-Armanet C (2012). Horizontal gene transfer of a chloroplast DnaJ-Fer protein to Thaumarchaeota and the evolutionary history of the DnaK chaperone system in Archaea. BMC Evol Biol.

[CR38] Prasad BD, Goel S, Krishna P (2010). *In silico* identification of carboxylate clamp type tetratricopeptide repeat proteins in *Arabidopsis* and rice as putative co-chaperones of Hsp90/Hsp70. PLoS One.

[CR39] Reeves R, Beckerbauer L (2001). HMGI/Y proteins: flexible regulators of transcription and chromatin structure. Biochim Biophys Acta.

[CR40] Ren X, Vorst O, Fiers M, Stiekema WJ, Nap J (2006). In plants, highly expressed genes are the least compact. Trends Genet.

[CR41] Richly H, Rocha-Viegas L, Ribeiro JD, Demajo S, Gundem G, Lopez-Bigas N, Nakagawa T, Rospert S, Takashi Ito, Croce LD (2010). Transcriptional activation of polycomb-repressed genes by ZRF1. Nature.

[CR42] Salas-Muñoz S, Rodríguez-Hernández AA, Ortega-Amaro MA, Salazar-Badillo FB, Jiménez-Bremont JF (2016). Arabidopsis AtDjA3 null mutant shows increased sensitivity to abscisic acid, salt, and osmotic stress in germination and post-germination stages. Front Plant Sci.

[CR43] Sarka NK, Thapar U, Kundnani PK, Panwar P, Grover A (2013). Functional relevance of J-protein family of rice (*Oryza sativa*). Cell Stress Chaperon.

[CR44] Sato Y, Antonio BA, Namiki N, Takehisa H, Minami H (2011). RiceXPro: a platform for monitoring gene expression in japonica rice grown under natural field conditions. Nucleic Acids Res.

[CR45] Servas C, Romisch K (2013). The Sec63p J-domain is required for ERAD of soluble proteins in yeast. PLoS One.

[CR46] Sery A, Housset D, Serre L, Bonicel J, Hatchikian C, Frey M, Roth M (1994). Crystal structure of the ferredoxin I from Desulfovibrio africanus at 2.3 Å resolution. Biochemistry.

[CR47] Shen L, Kang YGG, Liu L, Yu H (2011). The J-domain protein J3 mediates the integration of flowering signals in *Arabidopsis*. Plant Cell.

[CR48] Shiber A, Ravid T (2014). Chaperoning proteins for destruction: diverse roles of Hsp70 chaperones and their co-chaperones in targeting misfolded proteins to the proteasome. Biomolecules.

[CR49] Sielaff B, Tsai FTF (2010). The M-domain controls Hsp104 protein remodeling activity in an Hsp70/Hsp40-dependent manner. J Mol Biol.

[CR50] Sjögren L, Floris M, Barghetti A, Völlmy F, Linding R, Brodersen P (2018). Farnesylated heat shock protein 40 is a component of membrane-bound RISC in Arabidopsis. J Biol Chem.

[CR51] Sun J, Zhang J, Wu F, Xu C, Li S, Zhao W, Wu ZY, Wu JH, Zhou CZ, Shi YY (2005). Solution structure of Kti11p from *Saccharomyces cerevisiae* reveals a novel zinc-binding module. Biochemistry.

[CR52] Tamadaddi CA, Sahi C (2016). J domain independent functions of J proteins. Cell Stress Chaperon.

[CR53] Tamura K, Stecher G, Peterson D, Filipski A, Kumar S (2013). MEGA6: molecular evolutionary genetics analysis version 6.0. Mol Biol Evol.

[CR54] Valencia-Morales MD, Camas-Reyes JA, Cabrera-Ponce JL, Alvarez-Venegas R (2012). The *Arabidopsis thaliana* SET-domain-containing protein ASHH1/SDG26 interacts with itself and with distinct histone lysine methyltransferases. J Plant Res.

[CR55] Verma AK, Diwan D, Raut S, Dobriyal N, Brown RE, Gowda V, Hines JK, Sahi C (2017). Evolutionary conservation and emerging functional diversity of the cytosolic Hsp70: J Protein Chaperone Network of *Arabidopsis thaliana*. G3-Genes Genome Genet.

[CR56] Voorrips RE (2002). Mapchart: software for the graphical presentation of linkage maps and QTLs. J Hered.

[CR57] Walsh P, Bursac D, Law YC, Cyr D, Lithgow T (2004). The J-protein family: modulating protein assembly, disassembly and translocation. EMBO J.

[CR58] Wang G, Cai G, Kong F, Deng Y, Ma N, Meng Q (2014). Overexpression of tomato chloroplast-targeted DnaJ protein enhances tolerance to drought stress and resistance to *Pseudomonas solanacearum* in transgenic tobacco. Plant Physiol Biochem.

[CR59] Wang GD, Kong FY, Zhang S, Meng X, Wang Y, Meng QW (2015). A tomato chloroplast-targeted DnaJ protein protects Rubisco activity under heat stress. J Exp Bot.

[CR60] Wang J, Wang JZ, Lu YZ, Fang Y, Gao X, Wang ZH, Zheng WJ, Xu SB (2018). The heat responsive wheat *TaRAD23* rescues developmental and thermotolerant defects of the *rad23b* mutant in *Arabidopsis thaliana*. Plant Sci.

[CR61] Wang GD, Cai GH, Xu N, Zhang LT, Sun XL, Guan J, Meng QW (2019). Novel DnaJ protein facilitates Thermotolerance of transgenic tomatoes. Int J Mol Sci.

[CR62] Xia ZL, Zhang XQ, Li JQ, Su XH, Liu JJ (2014). Overexpression of a tobacco J-domain protein enhance drought tolerance in transgenic *Arabidopsis*. Plant Physiol Biochem.

[CR63] Xu GX, Guo CC, Shan HY, Kong HZ (2012). Divergence of duplicate genes in exon-intron structure. Proc Natl Acad Sci USA.

[CR64] Yamamoto H, Peng L, Fukao Y, Shikanai T (2011). An Src homology 3 domain-like fold protein forms a ferredoxin binding site for the chloroplast NADH dehydrogenase-like complex in *Arabidopsis*. Plant Cell.

[CR65] Yang KZ, Xia C, Liu XL, Dou XY, Wang W, Chen LQ, Zhang XQ, Xie LF, He L, Ma X, Ye D (2009). A mutation in *thermosensitive male sterile 1*, encoding a heat shock protein with DnaJ and PDI domains, leads to thermosensitive gametophytic male sterility in *Arabidopsis*. Plant J.

[CR66] Zhang B, Qiu H, Qu D, Ruan Y, Chen DH (2018). Phylogeny-dominant classification of J-proteins in *Arabidopsis thaliana* and *Brassica oleracea*. Génome.

[CR67] Zhao ZC, Zhang WR, Yan JP, Zhang JJ, Liu Z, Li XF, Yi Y (2010). Over-expression of Arabidopsis DnaJ (Hsp40) contributes to NaCl-stress tolerance. AJB.

[CR68] Zhao XM, Leng YJ, Chen GX, Zhao PM, Ye D, Chen LQ (2015). The thermosensitive male sterile 1 interacts with the BiPs via DnaJ domain and stimulates their ATPase enzyme activities in Arabidopsis. PLoS One.

[CR69] Zhou W, Zhou T, Li MX, Zhao CL, Jia N, Wang XX, Sun YZ, Li GL, Xu M, Zhou RG, Li B (2012). The *Arabidopsis* J-protein AtDjB1 facilitates thermotolerance by protecting cells against heat-induced oxidative damage. New Phytol.

[CR70] Zhu JK, Shi J, Bressan RA, Hasegawa PM (1993). Expression of an Atriplex nummularia gene encoding a protein homologous to the bacterial molecular chaperone DnaJ. Plant Cell.

