# Supplementary material for: Genome-wide analysis of the rice J-protein family: identification, genomic organization, and expression profiles under multiple stresses
Source: 3 Biotech. 2019 Sep 6;9(10):358. doi: 10.1007/s13205-019-1880-8 (PMC6730974; doi:10.1007/s13205-019-1880-8)
Supplement: Supplementary file 1 — Supplementary material 1 (DOCX 68 kb). List of J-protein genes family in rice. Chr chromosome, AA number of amino acid, MW Molecular weight KDa, PI theoretical isoelectric point, DnaJ_CXXCXGXG zinc-finger domain, DnaJ_C C-terminal domain, DUF3444, DUF1997, DUF4339, DUF3395 and DUF3752 domain of function unknown, d1eq1a, d1mek and d1qqea domain of function unknown; HSCB_C C-terminal oligomerisation domain (heat shock cognate protein B), RRM RNA recognition motif, Fre4 ferredoxin domain [4Fe–4S] cluster, DnaJ-X X lies between the N-terminal DnaJ and the C-terminal Z domains, Sec63 named after the yeast Sec63, 3APQlB domain of function unknown, zf-CSL small protein motifs which contain multiple finger-like protrusions, AT_hook DNA-binding motifs with a preference for A/T rich regions, Jiv90 the region of a bovine J-protein Jiv interacting with viral polyprotein, TPR tetratricopeptide region, Up Up-regulation, Down Down-regulation, / represents the genes have no corresponding probe sets in the microarray data. *represents novel members of rice J-proteins [file 13205_2019_1880_MOESM1_ESM.docx]

**Supplementary Table 1** List of J-protein family genes in rice

| Gene ID | Chr | Genomic position | AA | MW (KDa) | PI | Additional domain | Heat  stress | Drought  stress | Salt  stress |
| --- | --- | --- | --- | --- | --- | --- | --- | --- | --- |
| Oligo-gene clades |  |  |  |  |  |  |  |  |  |
| Clade I |  |  |  |  |  |  |  |  |  |
| Os03g56540 | 3 | 32208880-32210854 | 97 | 10.93 | 11.33 |  | Up | Down | Down |
| Os01g06454 | 1 | 3044348-3041412 | 113 | 12.07 | 10.60 |  | Up | Up | Up |
| Os07g09450 | 7 | 4981656-4977967 | 113 | 12.030 | 10.73 |  | Up | Up | Up |
| Os03g54150 | 3 | 31027479-31032567 | 613 | 67.04 | 4.75 |  | Down | Up | Up |
| * Os12g31460 | 12 | 18920690-18918230 | 236 | 25.21 | 4.53 |  | Up | Down | Down |
| Os11g43950 | 11 | 26542837-26549304 | 889 | 96.86 | 5.13 | d1eq1a | Down | Down | Up |
| Os12g36180 | 12 | 22180501-22173282 | 926 | 10.20 | 6.74 |  | Down | Up | Up |
| Os01g25320 | 1 | 14303222-14292067 | 949 | 104.27 | 4.75 | d1eq1a | Up | Up | Up |
| Os05g50370 | 5 | 28864052-28871102 | 1424 | 157.29 | 5.02 |  | Down | Down | Down |
| Os01g44310 | 1 | 25425723-25417248 | 1473 | 163.18 | 4.95 |  | Up | Up | Up |
| Os03g10180 | 3 | 5161510-5166769 | 608 | 66.47 | 5.62 |  | Down | Down | Down |
| Clade III |  |  |  |  |  |  |  |  |  |
| Os01g01160 | 1 | 82428-84302 | 191 | 21.44 | 10.14 |  | Down | Down | Down |
| Os08g43490 | 8 | 27507617-27508511 | 147 | 15.73 | 10.82 |  | Down | Down | Down |
| * Os01g70250 | 1 | 40677841-40674891 | 761 | 86.06 | 5.66 | DUF3444 | Up | Up | Up |
| Os01g53020 | 1 | 30472593-30475205 | 343 | 38.11 | 8.34 | Fre4_13 | Up | Down | Down |
| Os05g45350 | 5 | 26314173-26311382 | 351 | 39.14 | 6.30 | Fre4 | Down | Down | Down |
| Os04g57880 | 4 | 34489919-34492725 | 487 | 52.58 | 9.62 | Fre4_13 | Up | Up | Up |
| Os07g43330 | 7 | 25940608-25943189 | 271 | 31.19 | 9.97 |  | Down | Down | Down |
| Os03g20730 | 3 | 11731439-11730942 | 166 | 17.91 | 11.04 |  | Down | Down | Down |
| Os09g20320 | 9 | 12190875-12193475 | 330 | 36.24 | 10.22 |  | / | / | / |
| Clade IV |  |  |  |  |  |  |  |  |  |
| Os02g46640 | 2 | 28467362-28464489 | 122 | 14.21 | 6.50 |  | Down | Up | Up |
| Os02g52270 | 2 | 32003856-32001646 | 133 | 15.54 | 5.05 |  | Up | Down | Down |
| Os09g32050 | 9 | 19129934-19125390 | 396 | 44.17 | 4.87 | DnaJ-X | Up | Up | Up |
| Os08g41110 | 8 | 25979471-25984017 | 395 | 44.23 | 5.34 | DnaJ-X | Down | Up | Up |
| Os02g35000 | 2 | 20997195-20994336 | 378 | 42.48 | 5.25 | DnaJ-X | Up | Down | Down |
| Os05g46620 | 5 | 26995161-26991242 | 339 | 37.90 | 6.53 | DnaJ-X | Up | Down | Up |
| Os01g50700 | 1 | 29119761-29124636 | 653 | 71.04 | 7.59 | DnaJ-X, Dehydrin | Down | Down | Down |
| Os01g42190 | 1 | 23911517-23913299 | 198 | 21.67 | 5.27 |  | Up | Up | Up |
| Os03g18870 | 3 | 10573269-10571639 | 167 | 17.99 | 5.96 |  | Up | Up | Up |
| Os08g35160 | 8 | 22159508-22161430 | 159 | 16.73 | 5.31 |  | Up | Up | Down |
| Os06g09560 | 6 | 4867843-4870168 | 236 | 24.53 | 4.23 |  | Up | Down | Up |
| Os02g54130 | 2 | 33175612-33169843 | 272 | 29.25 | 6.04 |  | Down | Up | Up |
| Os01g32870 | 1 | 18041759-18050240 | 404 | 44.95 | 6.11 |  | Up | Up | Up |
| Os02g50760 | 2 | 30994374-30998999 | 443 | 49.32 | 9.26 |  | Down | Up | Up |
| Os06g13060 | 6 | 7164156-7159988 | 436 | 49.13 | 8.80 |  | Up | Up | Up |
| Clade IX |  |  |  |  |  |  |  |  |  |
| Os12g41820 | 12 | 25907573-25901456 | 545 | 61.40 | 8.67 | Jiv90 | Up | Up | Up |
| Os03g61730 | 3 | 34997730-34991850 | 726 | 80.04 | 9.43 | Jiv90 | Up | Up | Up |
| * Os03g19200 | 3 | 10775798-10772280 | 669 | 70.56 | 10.25 | TPR_1 | Down | Up | Down |
| * Os07g49000 | 7 | 29338447-29341163 | 604 | 64.18 | 8.62 | d1qqea | Down | Down | Up |
| Os05g31062 | 5 | 18052572-18056393 | 395 | 44.43 | 8.04 | TPR | Up | Up | Up |
| Os01g74580 | 1 | 43189156-43193534 | 472 | 52.83 | 6.91 | TPR | Up | Up | Down |
| Os02g10180 | 2 | 5336973-5331895 | 477 | 53.27 | 7.15 | TPR | Up | Up | Up |
| Multigene clades |  |  |  |  |  |  |  |  |  |
| Clade VI |  |  |  |  |  |  |  |  |  |
| Os03g12236 | 3 | 6421734-6426920 | 257 | 28.11 | 9.05 |  | Up | Up | Down |
| Os05g26902 | 5 | 15622604-15617643 | 448 | 48.21 | 10.15 | DnaJ_CXXCXGXG | Up | Up | Up |
| Os05g26926 | 5 | 15638497-15635360 | 448 | 48.21 | 10.15 | DnaJ_CXXCXGXG | Up | Up | Up |
| Os02g56040 | 2 | 34307729-34300624 | 488 | 52.01 | 9.18 | DnaJ_CXXCXGXG | Up | Up | Down |
| Os06g02620 | 6 | 932583-927338 | 443 | 47.37 | 9.93 | DnaJ_CXXCXGXG | Up | Down | Down |
| Os12g07060 | 12 | 3455589-3463158 | 420 | 45.69 | 8.58 | DnaJ_CXXCXGXG | Up | Up | Up |
| Os06g11440 | 6 | 6050429-6036698 | 1294 | 145.26 | 8.14 | DnaJ_CXXCXGXG | Up | Up | Up |
| Os04g46390 | 4 | 27505490-27510048 | 417 | 47.14 | 7.00 | DnaJ_CXXCXGXG | Down | Down | Down |
| Os02g43930 | 2 | 26519326-26523437 | 422 | 47.23 | 7.35 | DnaJ_CXXCXGXG | Down | Up | Up |
| Os03g57340 | 3 | 32708411-32711331 | 418 | 46.69 | 6.08 | DnaJ_CXXCXGXG | Up | Down | Down |
| Os03g44620 | 3 | 25127234-25131074 | 418 | 46.48 | 6.36 | DnaJ_CXXCXGXG | Down | Up | Up |
| Os12g42440 | 12 | 26377868-26379849 | 468 | 49.39 | 4.86 | DnaJ_CXXCXGXG | / | / | / |
| Os01g13760 | 1 | 7712339-7721253 | 350 | 38.53 | 9.58 | DnaJ_C | Down | Down | Down |
| Os02g20394 | 2 | 12019009-12023447 | 350 | 38.53 | 9.08 | DnaJ_C | / | / | / |
| Os05g48810 | 5 | 27974607-27972291 | 363 | 38.67 | 9.95 | DnaJ_C | Up | Down | Down |
| Os05g03630 | 5 | 1553433-1557251 | 323 | 35.02 | 9.90 | DnaJ_C | / | / | / |
| Os02g03600 | 2 | 1474710-1468977 | 390 | 42.99 | 9.47 | DnaJ_C | Down | Down | Down |
| Os08g06460 | 8 | 3624999-3622704 | 343 | 38.20 | 9.98 | DnaJ_C | Down | Up | Up |
| Os08g28700 | 8 | 17540911-17538199 | 345 | 37.05 | 5.31 | DnaJ_C | Down | Up | Up |
| Os01g65480 | 1 | 38009626-38012278 | 328 | 36.05 | 9.56 | DnaJ_C | Down | Up | Down |
| Os05g06440 | 5 | 3303197-3307713 | 348 | 39.27 | 6.88 | DnaJ_C | Up | Up | Up |
| * Os07g32950 | 7 | 19692304-19695407 | 527 | 61.36 | 6.97 | DnaJ_C | Up | Down | Up |
| * Os07g43870 | 7 | 26230679-26240915 | 689 | 77.85 | 6.29 | d1mek | Up | Up | Up |
| Os08g36980 | 8 | 23386887-23388722 | 175 | 19.29 | 4.39 | zf-CSL | Down | Up | Up |
| Os09g28590 | 9 | 17393569-17395622 | 197 | 22.12 | 4.57 | zf-CSL | Up | Up | Up |
| Clade VII |  |  |  |  |  |  |  |  |  |
| Os03g62150 | 3 | 35207543-35208331 | 263 | 28.24 | 8.69 |  | / | / | / |
| Os08g37270 | 8 | 23557556-23559107 | 397 | 41.57 | 9.83 | AT_hook | / | / | / |
| Os09g28890 | 9 | 17556929-17558591 | 373 | 39.49 | 8.63 | AT_hook | Up | Up | Down |
| * Os07g42800 | 7 | 25636703-25639894 | 397 | 41.41 | 6.97 |  | Up | Up | Up |
| * Os03g27460 | 3 | 15756106-15751337 | 406 | 42.38 | 6.90 |  | Down | Up | Up |
| Os11g37000 | 11 | 21839186-21843540 | 625 | 69.49 | 6.09 |  | / | / | / |
| Os01g27740 | 1 | 15478322-15474284 | 1009 | 113.41 | 8.19 | DUF3444 | Up | Up | Down |
| Os11g36960 | 11 | 21806099-21809530 | 1053 | 119.02 | 6.80 | DUF3444 | Up | Up | Down |
| Os06g34440 | 6 | 20042684-20034666 | 1019 | 113.28 | 6.84 | DUF3444 | Down | Up | Down |
| Os04g31940 | 4 | 19130179-19133059 | 730 | 81.73 | 9.23 | DUF3444 | Down | Up | Up |
| Os02g30620 | 2 | 18227411-18230654 | 735 | 82.39 | 9.24 | DUF3444 | Down | Up | Up |
| Os03g28310 | 3 | 16297124-16293645 | 749 | 83.40 | 7.73 | DUF3444 | Up | Up | Up |
| Os01g69930 | 1 | 40438233-40442444 | 745 | 83.44 | 9.12 | DUF3444 | / | / | / |
| Os01g37560 | 1 | 20987760-20992534 | 381 | 42.74 | 8.20 | DUF1977 | Up | Up | Up |
| Os05g30130 | 5 | 17445927-17441559 | 368 | 42.59 | 9.77 | DUF1977 | Down | Up | Up |
| * Os12g44260 | 12 | 27442429-27441760 | 163 | 17.03 | 9.36 |  | / | / | / |
| Os03g62130 | 3 | 35202558-35203388 | 277 | 29.78 | 8.64 |  | / | / | / |
| Os03g62140 | 3 | 35205727-35206590 | 288 | 30.86 | 8.49 |  | / | / | / |
| Os03g62120 | 3 | 35198684-35201378 | 478 | 50.23 | 10.14 |  | / | / | / |
| Os10g11012 | 10 | 6097287-6093142 | 374 | 40.06 | 7.50 |  | / | / |  |
| Os10g03610 | 10 | 1561403-1562164 | 254 | 27.56 | 10.83 |  | / | / |  |
| Os03g36160 | 3 | 20061677-20062555 | 293 | 31.71 | 9.83 |  | / | / |  |
| Os11g36530 | 11 | 21556427-21552630 | 291 | 31.81 | 8.07 |  | / | / | / |
| Os03g61550 | 3 | 34904864-34906089 | 261 | 28.63 | 10.75 |  | / | / | / |
| Mono-gene clades |  |  |  |  |  |  |  |  |  |
| Clade II |  |  |  |  |  |  |  |  |  |
| Os12g27070 | 12 | 15851558-15848618 | 261 | 29.05 | 8.86 | HSCB_C | Down | Down | Down |
| * Os08g03380 | 8 | 1584989-1580877 | 294 | 32.46 | 9.82 |  | Up | Up | Up |
| * Os10g33790 | 10 | 17910800-17905866 | 299 | 32.61 | 9.92 |  | Up | Down | Up |
| Os03g04400 | 3 | 2022080-2018676 | 297 | 32.30 | 9.50 | RRM | Down | Up | Up |
| Clade V |  |  |  |  |  |  |  |  |  |
| Os01g17030 | 1 | 9754542-9755797 | 151 | 15.66 | 10.53 |  | / | / | / |
| Os01g17040 | 1 | 9764973-9766481 | 212 | 22.95 | 9.84 |  | / | / | / |
| Os10g36370 | 10 | 19439780-19430957 | 541 | 60.14 | 8.93 | DUF3395 | Up | Up | Up |
| Os03g55360 | 3 | 31495921-31500606 | 506 | 57.03 | 7.91 |  | Up | Up | Up |
| Os07g44310 | 7 | 26471576-26473120 | 135 | 14.94 | 10.86 |  | Down | Down | Down |
| Os12g31840 | 12 | 19158097-19161631 | 608 | 68.21 | 5.47 | DnaJ_CXXCXGXG | Down | Up | Up |
| Os06g44160 | 6 | 26647896-26646324 | 143 | 16.09 | 6.94 |  | Down | Down | Up |
| Os03g60790 | 3 | 34539517-34541043 | 269 | 29.65 | 7.73 |  | Up | Down | Down |
| Os04g24180 | 4 | 13834743-13841261 | 682 | 76.24 | 5.96 | Sec63 | Up | Up | Up |
| Os03g15480 | 3 | 8501364-8505676 | 299 | 34.56 | 10.19 |  | Down | Up | Up |
| Os01g33800 | 1 | 18600976-18611575 | 604 | 67.63 | 9.76 | DUF3752 | Up | Down | Down |
| Os02g10220 | 2 | 5351467-5355147 | 283 | 32.45 | 7.43 |  | Up | Up | Up |
| Os12g15590 | 12 | 8915106-8906426 | 310 | 36.16 | 9.68 |  | Up | Up | Up |
| Os03g18200 | 3 | 10204819-10210306 | 664 | 72.12 | 9.87 | 3APQlB | Down | Down | Down |
| Clade VIII |  |  |  |  |  |  |  |  |  |
| Os04g59060 | 4 | 35132608-35128585 | 275 | 31.35 | 10.29 |  | Up | Up | Down |
| Os05g01590 | 5 | 351640-354043 | 231 | 26.20 | 9.87 |  | Up | Up | Down |
| Os07g28800 | 7 | 16867122-16863279 | 270 | 31.41 | 9.14 |  | Up | Up | Up |
| Os10g42439 | 10 | 22875955-22861341 | 2633 | 287.69 | 6.36 | DUF4339, ARM | Up | Up | Up |
| Os03g51830 | 3 | 29724283-29729904 | 239 | 28.06 | 10.16 |  | Up | Up | Up |
| Os07g03270 | 7 | 23557556-23559107 | 397 | 41.57 | 9.83 |  | Down | Up | Up |

Chr chromosome, AA number of amino acid, MW Molecular weight KDa, PI theoretical isoelectric point, DnaJ_CXXCXGXG zinc finger domain, DnaJ_C C-terminal domain, DUF3444, DUF1997, DUF4339, DUF3395 and DUF3752 domain of function unknown, d1eq1a, d1mek and d1qqea domain of function unknown; HSCB_C C-terminal oligomerisation domain (heat shock cognate protein B), RRM RNA recognition motif, Fre4 ferredoxin domain [4Fe-4S] cluster, DnaJ-X X lies between the N-terminal DnaJ and the C-terminal Z domains, Sec63 named after the yeast Sec63, 3APQlB domain of function unknown, zf-CSL small protein motifs which contain multiple finger-like protrusions, AT_hook DNA-binding motifs with a preference for A/T rich regions, Jiv90 the region of a bovine J-protein Jiv interacting with viral polyprotein, TPR tetratricopeptide region, Up Up-regulation, Down Down-regulation, / represents the genes have no corresponding probe sets in the microarray data. * represents novel members of rice J-proteins.
